# Effect of Negative Pressure on Proliferation, Virulence Factor Secretion, Biofilm Formation, and Virulence-Regulated Gene Expression of* Pseudomonas aeruginosa* In Vitro

**DOI:** 10.1155/2016/7986234

**Published:** 2016-12-15

**Authors:** Guo-Qi Wang, Tong-Tong Li, Zhi-Rui Li, Li-Cheng Zhang, Li-Hai Zhang, Li Han, Pei-Fu Tang

**Affiliations:** ^1^Department of Orthopedics, Chinese PLA General Hospital, No. 28 Fuxing Road, Beijing 100853, China; ^2^Department of Orthopedics, Tianjin Hospital, No. 406 Jiefangnan Road, Tianjin 300211, China; ^3^Center for Hospital Infection Control, Chinese PLA Institute for Disease Control and Prevention, Beijing 100071, China

## Abstract

*Objective*. To investigate the effect of negative pressure conditions induced by NPWT on* P. aeruginosa*.* Methods*.* P. aeruginosa* was cultured in a Luria–Bertani medium at negative pressure of −125 mmHg for 24 h in the experimental group and at atmospheric pressure in the control group. The diameters of the colonies of* P. aeruginosa* were measured after 24 h. ELISA kit, orcinol method, and elastin-Congo red assay were used to quantify the virulence factors. Biofilm formation was observed by staining with Alexa Fluor® 647 conjugate of concanavalin A (Con A). Virulence-regulated genes were determined by quantitative RT-PCR.* Results*. As compared with the control group, growth of* P. aeruginosa* was inhibited by negative pressure. The colony size under negative pressure was significantly smaller in the experimental group than that in the controls (*p* < 0.01). Besides, reductions in the total amount of virulence factors were observed in the negative pressure group, including exotoxin A, rhamnolipid, and elastase. RT-PCR results revealed a significant inhibition in the expression level of virulence-regulated genes.* Conclusion*. Negative pressure could significantly inhibit the growth of* P. aeruginosa*. It led to a decrease in the virulence factor secretion, biofilm formation, and a reduction in the expression level of virulence-regulated genes.

## 1. Introduction

Infection is considered one of the most critical factors in impeding wound healing [1]. When the skin or tissue is compromised, bacteria can easily access the underlying tissues, which are believed to be the optimal places for colonization and growth of bacteria. It is reported that the infection rate was as much as 12% in acute wounds and 38% in chronic wounds [2], posing a challenge to clinical doctors.* Pseudomonas aeruginosa *(*P. aeruginosa*), a kind of gram-negative bacteria, is one of the most common pathogens isolated from wound infections [3]. It has been widely used in wound infection-related studies [4–6] owing to its virulence factor secretion and biofilm formation.* P. aeruginosa* can secrete various exotoxins, such as exotoxin A, rhamnolipid, and elastase, which play an important role in impeding wound healing and inflammatory reaction [7–9]. Moreover, exotoxin A and elastase are encoded by* ToxA* and* LasB* and the* RhlA* gene encodes a rhamnolipid synthase involved in the biosynthetic pathway [10, 11].* P. aeruginosa* expresses two types of quorum sensing (QS) systems,* LasI* and* RhlI*, which contribute to the pathology of cutaneous wound infections [12, 13]. Based on this fact, the search for measures to inhibit toxin production and biofilm formation is an active area of clinical research. Recently, as an effective management of contaminated wounds, negative pressure wound therapy (NPWT) has been widely used in clinical laboratories [14, 15]. However, whether NPWT could reduce the bacterial load of wounds is still controversial. Weed reported that bacterial colonization increased significantly with NPWT [16]. Lalliss found that NPWT showed a significant and sustained decrease in the* P. aeruginosa* levels compared to WTD dressings [17]. However, the mechanism underlying the action of NPWT in the reduction of* P. aeruginosa* levels is still unknown. It is well known that both the immune status of host and bacterial invasiveness play important roles in the infection process [18]. Thus, the mechanism explaining the change in* P. aeruginosa* levels could not be confirmed under NPWT in vivo. Besides, few studies have reported the bacteria in wounds, secondary to negative pressure treatment, particularly with regard to* P. aeruginosa* proliferation, virulence, and gene expression. Previous studies have indicated that negative pressure induced by NPWT could alter the gene expression and proliferation of bone marrow mesenchymal stem cells [19, 20]. Our previous work had shown that negative pressure had an effect on the growth, secretion, and biofilm formation of* Staphylococcus aureus* [21].

The aim of this study was to evaluate the influence of negative pressure on the proliferation, virulence factor secretion, biofilm formation, and the virulence-regulated gene expression of* P. aeruginosa* in vitro.

## 2. Materials and Methods

### 2.1. Bacterial Strain and Preparation

*P. aeruginosa* laboratory strain PAO1 carrying the gene encoding the green fluorescent protein (GFP) was obtained from the laboratory of the Chinese PLA Institute for Disease Control and Prevention (Beijing, China).* P. aeruginosa* was grown overnight and cultured in Luria broth at 37°C until log-phase was achieved. Optical density at 600 nm wavelength was measured. An optical density of 1.0 was equivalent to 10^5^ colony-forming units per microliter, as determined by a standard curve.

### 2.2. Growth Conditions

The bacterial culture protocol was based on our previously published model of in vitro negative pressure condition [21]. In brief, negative pressure condition was created for bacterial growth and an airtight chamber was used as the incubator. The air was sucked from the chamber by a vacuum pump device (provided by Professor Hu Lei, Beihang University, Beijing, China), which could automatically produce and maintain the negative pressure at −125 mmHg. The O_2_ concentration was constantly maintained at 20%, as adequate amount of room air was introduced into the incubator every 15 min. Bacterial culture was performed in culture dishes (Corning Life Sciences, USA) with a diameter of 35 mm at 37°C. Each of the dishes contained 2 mL LB medium and 10^6^ *P. aeruginosa *(in a volume of 10 *μ*L) was added. Bacteria in the control group were grown under atmospheric pressure, and other conditions were the same as that of the experimental group.

### 2.3. Morphological Characterization of Bacterial Colony

LB agar plates were inoculated with 2 *μ*L of bacterial culture (OD at 600 nm = 1.0).* P. aeruginosa *was grown under aforementioned culture conditions for 24 h. To evaluate the colonial morphology, including the shape, color, size, and surface, a digital camera (IXUSi, Canon, Japan) was used to capture images of the bacterial colonies. The colony diameter was independently measured by two observers and the results were averaged.

### 2.4. Growth Curves

Bacteria were grown in 2 mL LB broth, with an inoculation of 10^6^ *P. aeruginosa* in culture dishes at 37°C under a static condition. The growth of the bacteria exposed and unexposed to negative pressure was measured by reading the OD values at 600 nm after every 60 min with adequate mixing.

### 2.5. Virulence Factor Assays

Exotoxin A was measured according to the method of Shigematsu et al. [22] and was determined using a commercially available Human Pseudomonas Exotoxin A (PEA) ELISA Kit (Cusabio Biotech Co., Ltd., Hubei, PR China, product code: CSB-E11252 h), according to the manufacturer's instructions. The data were recorded as ng/mL.

Rhamnolipid was quantified by orcinol method, as previously described with a few modifications [23]. Briefly, 400 *μ*L supernatant from the bacterial culture was extracted twice using 600 *μ*L diethyl ether. The ether layer was transferred to a fresh tube for evaporation. Residues were dissolved in 150 *μ*L H_2_O, 100 *μ*L 1.6% orcinol (Sigma), and 750 *μ*L 60% sulphuric acid (H_2_SO_4_). After heating for 30 min at 80°C, all the tubes were cooled at room temperature for 30 min and absorbance was recorded at 421 nm. The concentrations of rhamnolipid were calculated by multiplying rhamnose values by a coefficient of 2.5, as previously described [24].

The elastase activity was measured by the elastin-Congo red assay, as previously described [23]. Briefly, 100 *μ*L supernatant from 24 h LB cultures was added to tubes containing 10 mg of elastin-Congo red (Sigma) and 900 *μ*L Na_2_HPO_4_ (pH 7.0). Tubes were incubated for 4 h at 37°C under shaking conditions and the absorbance was recorded at 495 nm after removing the precipitate by centrifugation.

### 2.6. Static Biofilm Assays

To observe the influence of negative pressure on biofilm formation, 18 × 18 mm cover glass was put into a 35 mm culture dish, and each dish was incubated with 2 mL LB broth containing 10^6^ *P. aeruginosa* for 24 h in a constant temperature incubator at 37°C. After 24 h, each cover glass was washed three times with phosphate buffered saline (PBS) to remove planktonic bacteria. The* P. aeruginosa* glycocalyx was visualized by staining with 50 *μ*g/mL of Alexa Fluor 647 conjugate of Con A (Life Technologies, USA) for 15 min at room temperature in the dark as previously described with a few modifications [4]. Biofilm formation was observed through fluorescence microscopy (Olympus BX51).

### 2.7. Quantitative RT-PCR

Bacteria were isolated from the LB medium for quantitative RT-PCR analysis as previously described [21]. Primers used to amplify* ToxA, RhlA, LasB*,* LasI*, and* RhlI,* as well as the reference gene,* RpoD*, are shown in Table 1. Briefly, total RNA was extracted using an RNAprep Pure Cell/Bacteria Kit (TIANGEN, China) according to the manufacturer's instructions. Total RNA was treated with Recombinant DNase I (TAKARA, Japan) and reverse-transcribed using the TIANScript RT Kit (TIANGEN) according to the manufacturer's instructions. Real-time PCR analyses using the SYBR FAST qPCR Kit Master Mix Universal (KAPA, USA) were performed with an ABI7900HT sequence detection system (ABI, USA). The reaction procedures were as follows: incubation at 95°C for 3 min, 40 cycles at 95°C for 3 s, 60°C for 20 s, and one dissociation step at 95°C for 15 s, 60°C for 15 s, and 95°C for 15 s. All samples were analyzed in triplicate and normalized against* RpoD* expression. Results were shown as the fold change of gene expression relative to the control.

### 2.8. Statistical Analysis

SPSS 17.0 was used for the statistical analysis. The measurement data were expressed as mean ± SD and compared between the two groups using Student's *t*-test. A *p* value less than 0.05 was considered to be statistically significant.

## 3. Results

### 3.1. Growth of* P. aeruginosa* under Negative Pressure

**Figure 1:**
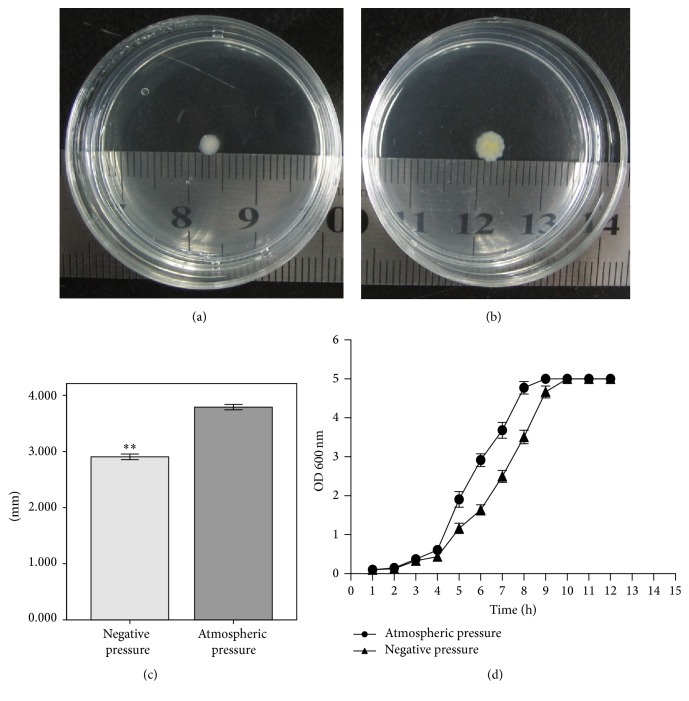
Colony of* P. aeruginosa *under negative pressure (a) and atmospheric pressure (b) at 24 h. (c) Diameters of colony of* P. aeruginosa *in two groups at 24 h, *N* = 10, ^*∗∗*^ *p* < 0.01. (d) Growth curve of* P. aeruginosa*. OD 600 nm value was recorded per hour (*N* = 3).

The diameters of the colonies of the two groups are shown in Figures 1(a) and 1(b). Colonies in both groups were round in shape. However, colonies under negative pressure were light in color. Moreover, the size of colonies under negative pressure was significantly smaller than that of the controls (*p* < 0.01, Figure 1(c)). The OD for the growth curve of* P. aeruginosa* was measured at 600 nm and it is shown in Figure 1(d). The growth rate of bacteria under negative pressure was less than that under atmospheric pressure from the third hour. Besides, the time to reach maximum OD (OD at 600 nm, 5.0) was 1 hour longer in the experimental group than that in the control group.

### 3.2. Effect of Negative Pressure on the Production of Virulence Factors

**Figure 2:**
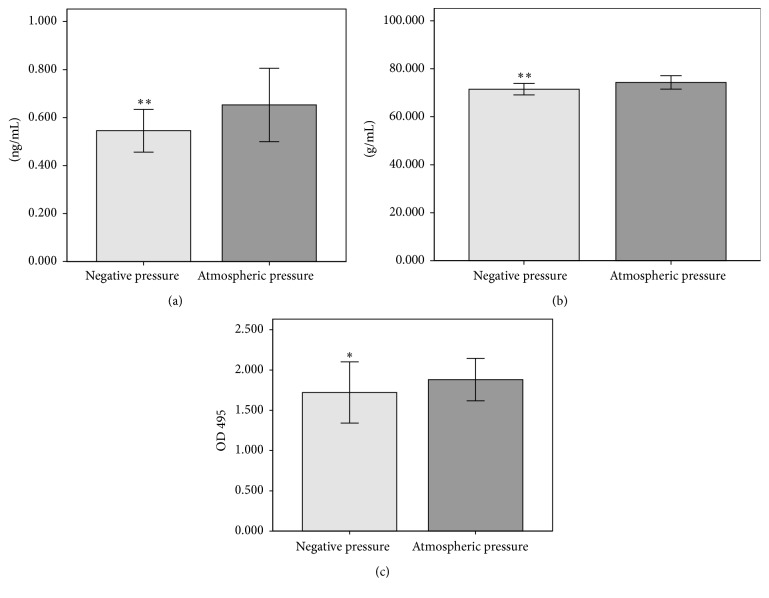
Detection of virulence factors following negative pressure and atmospheric pressure at 24 h. Production of exotoxin A (a), rhamnolipid (b), and elastase (c) in negative pressure group was significantly less than that in atmospheric pressure group. ^*∗*^ *p* < 0.05, ^*∗∗*^ *p* < 0.01, and *N* = 10.

Bacteria were cultured under negative pressure or atmospheric pressure for 24 h. The content of exotoxin A, rhamnolipid, and elastase secreted by* P. aeruginosa* was measured to evaluate the effect of negative pressure on the main virulence factors. Exotoxin A in the negative pressure group was significantly less than that in the control group (*p* < 0.01) (Figure 2(a)). A similar effect was observed for rhamnolipid and elastase (*p* < 0.01 and *p* < 0.05, resp.) (Figures 2(b) and 2(c)).

### 3.3. Biofilm Formation

**Figure 3:**
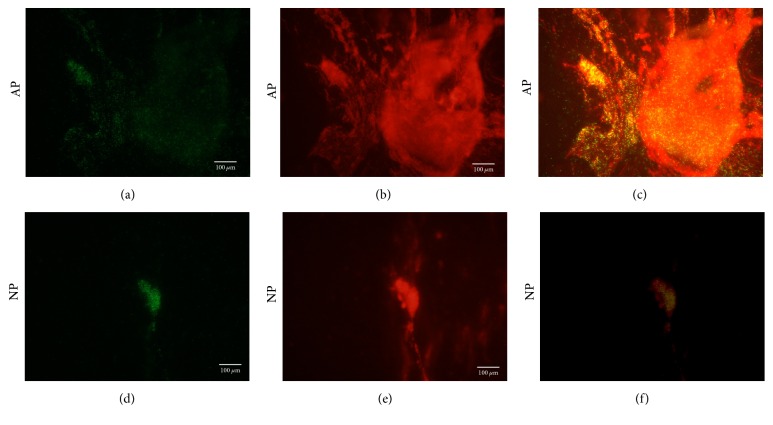
The* P. aeruginosa* glycocalyx was visualized by staining with 50 *μ*g/mL of Alexa Fluor 647 conjugate of Con A. Static biofilm assays under atmospheric pressure (AP) and negative pressure (NP) were shown.* P. aeruginosa* (green) under negative pressure conditions (d–f) were apt to be small aggregates and exhibited a reduced capacity for biofilm (red) adherence on the cover glass relative to the control (a–c) at 24 h.

Biofilm formation was observed in both the atmospheric pressure (AP) group and the negative pressure (NP) group through fluorescence microscopy at 24 h.* P. aeruginosa *(green) were observed to be big aggregates with excessive biofilm (red) under atmospheric pressure (Figures 3(a)–3(c)). However,* P. aeruginosa *(green) were observed to be small aggregates with a small amount of biofilm (red) under negative pressure (Figures 3(d)–3(f)).

### 3.4. Negative Pressure Changes Virulence and Biofilm-Regulated Genes in* P. aeruginosa*

**Figure 4:**
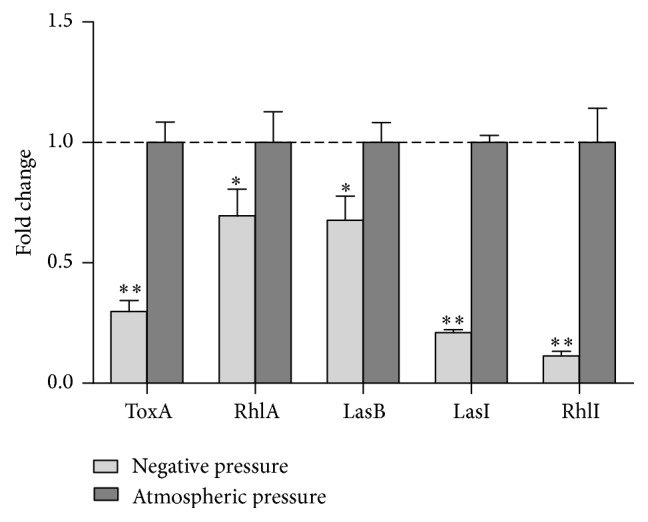
Analysis of virulence and biofilm-regulated genes,* ToxA, RhlA, LasB, LasI, *and* RhlI*, under negative pressure and atmospheric pressure. The atmospheric pressure group was used as calibrator with a value of 1, ^*∗*^ *p* < 0.05, ^*∗∗*^ *p* < 0.01, and *N* = 3. Expression of* ToxA, RhlA*,* LasB, LasI, *and* RhlI* in negative pressure group was 0.3-, 0.7-, 0.68-, 0.21-, and 0.11-fold that of the control group, respectively.

To investigate the mechanism of negative pressure induction in reducing virulence of* P. aeruginosa*, quantitative real-time PCR was used to assess relative expression levels of* ToxA, RhlA*,* LasB*,* LasI, *and* RhlI* genes. Negative pressure was found to significantly inhibit the transcription of* ToxA, RhlA*,* LasB*,* LasI*, and* RhlI* and the expression of these genes in the negative pressure group was 0.3-, 0.7-, 0.68-, 0.21-, 0.11-fold that of the control group, respectively (Figure 4). The repression of these genes under negative pressure supports the observed reduction in virulence factors and biofilm formation of* P. aeruginosa*.

## 4. Discussion

In recent years, physical therapies have been increasingly popular in the management of contaminated wounds owing to their satisfying wound closure and low risk of microbial resistance [25]. In particular, NPWT has been shown to promote the healing rates and prevent wound infections by multiple mechanisms, including decreasing edema, removal of wound exudates, and translating physical stimulation to signal transduction in cells [26, 27]. Previous studies indicated that negative pressure conditions caused by NPWT could alter the gene expression and the function of host cells in vitro, such as bone marrow MSCs and keratinocytes [19, 20, 28]. However, its potential effects on* P. aeruginosa* and virulence factors have not been studied yet. In this study, we investigated the effect of negative pressure on the proliferation, virulence factor secretion, and virulence-regulated gene expression of* P. aeruginosa*, which is one of the most frequently isolated pathogens during wound infections [3].

In this study, the negative pressure value (−125 mmHg) was consistent with the clinical use of negative pressure in NPWT, and the O_2_ tension was kept at 20% during bacterial culture in order to reduce interference from low oxygenation [29]. Colony diameter and growth curve indicated that negative pressure conditions could significantly inhibit the proliferation and growth rate of* P. aeruginosa.* Physical stimulations caused by pressure variation may contribute to this inhibition. Similarly, Liu et al. found that NPWT could decrease proliferation of* P. aeruginosa* within the burn wound and reduce mortality in a murine model [6]. Previous studies have found that physical stimulations, such as shear stress and hydrostatic pressure, could decrease the growth rate of* S. aureus*, attenuate bacterial virulence, and increase susceptibility to antimicrobial treatment [30, 31]. Furthermore, significant decrease in metabolic functions, such as carbohydrate metabolism and protein synthesis, was also observed under shear stress conditions [32]. Thus, it is hypothesized that negative pressure might inhibit the growth of* P. aeruginosa* by altering the metabolic rate.

Exotoxin A, rhamnolipid, and elastase are the main virulence factors secreted by* P. aeruginosa*, which play an important role in impeding wound healing and inflammatory reaction. It was reported that exotoxin A-producing strains showed a 20-fold increase in virulence in a murine model compared with exotoxin A-deficient mutants [33]. Rhamnolipid is known for its heat-stable extracellular hemolytic properties [34]. Elastase-producing* P. aeruginosa* isolates have been shown to significantly degrade human wound fluid as well as human skin proteins ex vivo [9]. Detection of these virulence factors indicated that they could be inhibited by negative pressure. One previous study has found that NPWT could evacuate toxins and exudates with the fluids from the wounds, which is one of its primary mechanisms [27]. However, results in our study might provide another promising explanation for NPWT in removing toxins from the wounds. Biofilm formation was supposed to be the key factor in resulting chronic infection [12, 35]. Our results indicated that* P. aeruginosa* tended to gather in small aggregates with a few biofilms under negative pressure, as compared to that under atmospheric pressure. In order to further investigate the mechanism of reduction in virulence factors and biofilm formation under negative pressure, the expression of virulence-regulated genes was analyzed. Results showed that these genes were repressed by negative pressure, which supported the observed reduction in virulence factors and biofilm formation of* P. aeruginosa*. Therefore, the influence of negative pressure on the production of exotoxin A, rhamnolipid, and elastase and biofilm formation might mainly depend on the inhibition of the* ToxA, RhlA*,* LasB*,* LasI*, and* RhlI *genes. This study has some limitations. First, all detections were carried out at 24 h after interventions. No long-term observation was available because the bacterial growth was inhibited by limited culture medium. Besides, only three virulence factors and five regulatory genes associated with wound infections were investigated in this study. As* P. aeruginosa* secretes several virulence factors, it is necessary to explore other toxins and regulatory genes in the future.

In conclusion, negative pressure could significantly inhibit the growth of* P. aeruginosa*. It also led to a decrease in the virulence factor secretion, biofilm formation, and a reduction in the expression level of virulence-regulated genes. This study indicated that a topical negative pressure condition, such as that used in NPWT, has the potential to be a novel anti-infection strategy to prevent and treat wound infections caused by* P. aeruginosa*.

## Figures and Tables

**Table 1:** Primer sequences for quantitative RT-PCR.

Gene	Primer	Amplicon (bp)
ToxA	Forward: GCCGATCTACACCATCGAGA Reverse: CATCTCGTTGCTCTCGTGC	94
RhlA	Forward: TGATCACCAAGGACGACGAG Reverse: GCCAGCAGCGTGGAGATAC	106
LasB	Forward: GACCCACAAGCTGTACATGAAG Reverse: CCAGCGGATAGAACATGGTG	110
LasI	Forward: ACTCAGCCGTTTCGCCAT Reverse: TCATCTTCTCCACGCCTACG	152
RhlI	Forward: ATTCTGGTCCAGCCTGCAA Reverse: CTGGAGGATCACGCCGTT	109
RpoD	Forward: AGAGAAGGACGACGAGGAAGAAG Reverse: GGCCAGGCCGGTGAGTTC	193
